# Macrophage modulation of dental pulp stem cell activity during tertiary dentinogenesis

**DOI:** 10.1038/s41598-020-77161-4

**Published:** 2020-11-19

**Authors:** Vitor C. M. Neves, Val Yianni, Paul T. Sharpe

**Affiliations:** 1 Centre for Craniofacial and Regenerative Biology, Faculty of Dentistry, Oral and Craniofacial Sciences, Kings College London, Floor 27, Guy’s Tower, Guy’s Hospital, London, SE1 9RT UK; 2 Centre for Host-Microbiome Interactions, Faculty of Dentistry, Oral and Craniofacial Sciences, Kings College London, London, UK

**Keywords:** Stem cells, Adult stem cells, Mesenchymal stem cells

## Abstract

The interaction between immune cells and stem cells is important during tissue repair. Macrophages have been described as being crucial for limb regeneration and in certain circumstances have been shown to affect stem cell differentiation in vivo. Dentine is susceptible to damage as a result of caries, pulp infection and inflammation all of which are major problems in tooth restoration. Characterising the interplay between immune cells and stem cells is crucial to understand how to improve natural repair mechanisms. In this study, we used an in vivo damage model, associated with a macrophage and neutrophil depletion model to investigate the role of immune cells in reparative dentine formation. In addition, we investigated the effect of elevating the Wnt/β-catenin pathway to understand how this might regulate macrophages and impact upon Wnt receiving pulp stem cells during repair. Our results show that macrophages are required for dental pulp stem cell activation and appropriate reparative dentine formation. In addition, pharmacological stimulation of the Wnt/β-catenin pathway via GSK-3β inhibitor small molecules polarises macrophages to an anti-inflammatory state faster than inert calcium silicate-based materials thereby accelerating stem cell activation and repair. Wnt/β-catenin signalling thus has a dual role in promoting reparative dentine formation by activating pulp stem cells and promoting an anti-inflammatory macrophage response.

## Introduction

In situations where there is substantial damage to the tooth structure leading to exposure of the dental pulp, the immune system is activated, and multiple immune cell types play a role in protecting the dental pulp against foreign bodies that can potentially lead to infection^1–6^. Macrophages have been reported to play important roles in tissue repair and regeneration by exerting their effects in a variety of ways during these processes^7^. Current research shows that depletion of macrophages is detrimental for muscle repair and limb regeneration^8–10^. Moreover, resident macrophages are described as regulators of inflammation levels by ‘cloaking’ microinjuries and regulating neutrophil recruitment^11,12^.

While it is known that macrophages are present in the dental pulp, their role in reparative dentine formation is not fully understood^13–15^. Recently, macrophage blockade was shown to directly affect Wnt receiving stem cell differentiation in the gut crypts, suggesting a possible direct interaction of macrophages and stem cells^16^. Understanding the inflammatory processes taking place in dental pulp in response to dentine damage, and the effects on local stem cell activation is important for designing therapies that can modulate tertiary dentine formation.

Macrophages are plastic cells that are classified into two main categories according to their physiological characteristics: pro-inflammatory classically activated (M1) or anti-inflammatory alternatively activated (M2)^17,18^. In kidney injury, M1 pro-inflammatory macrophages predominate in early phases of the injury, but the infiltrate present during the tissue repair phase has a predominantly M2 phenotype, suggesting that the timing of the M1–M2 transition is critical for tissue regeneration^19^.

Previously, we demonstrated that enhanced activation of the Wnt/β-catenin pathway stimulates pulp stem cell activity leading to a natural self-repair of deep dental lesions within the exposed dental pulp^20^. Here we used a small molecule GSK-3β antagonist (BIO, Merck), proven to enhance reparative dentine secretion via Wnt/β-catenin pathway activation, and compared it with a commonly used direct capping material (MTA, ProRoot Dentsply), to evaluate the biological differences between biological and inert direct capping in dental pulp immune reactions.

We used an in vivo dental pulp exposure model to investigate the role of macrophages in reparative dentine formation using a macrophage and neutrophil depletion model. We show that modification of immune cell responses in the dental pulp alters stem cell activation and dentine repair capacity. In addition, Wnt activation using GSK-3β inhibitor small molecules promotes macrophage polarisation into an anti-inflammatory state early in dental pulp repair. The pharmacological activation of Wnt/β-catenin pathway therefore plays a dual role in biological host modulation. It activates pulp stem cells and promotes anti-inflammatory macrophages, thereby demonstrating its relevance in clinical applications.

## Results

### Macrophages in reparative dentine formation

Histological analysis of macrophage localisation during odontogenesis in adult mouse molars shows that macrophages are present in the dental organ from development through to adulthood (Supplementary Fig. 1A–C). Moreover, in adult mouse molars, we observed that following dental pulp damage, an increasing number of macrophages populate the damage site during the reparative window (Supplementary Fig. 1D–I).

**Figure 1:**
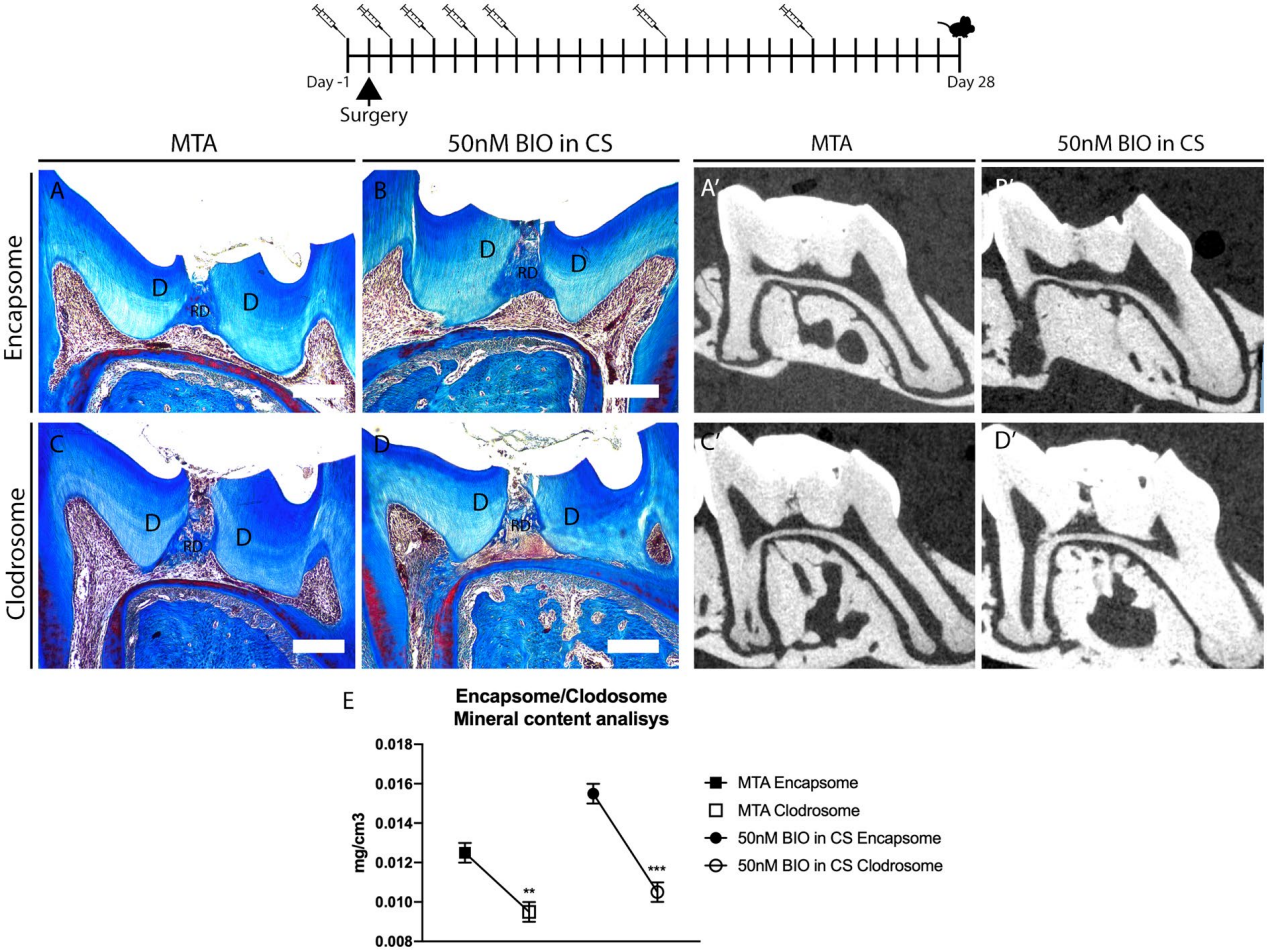
Macrophage reduction impairs reparative dentine secretion. Clodrosome and Encapsome injection time frame. Injections (syringe) and collection date (mouse). (**A**–**D**) Masson trichrome staining of molars capped with either MTA or 50 nM BIO in CS post 4 weeks of damage having gone through either Clodrosome or Encapsome injections. Mice injected with Encapsome showed normal repair in all treatments (MTA and 50 nM BIO in CS). The reparative dentine of mice injected with Clodrosome was affected as both MTA and 50 nM BIO in CS capping showed sparse reparative dentine secreted. (**A′**–**D′**) μCT Cross section image confirming through radiological image the difference of reparative dentine secretion between molars that had Encapsome injections and Clodrosome injections. (**E**) μCT mineral content analysis on the damage site showed significant decrease of mineral in MTA and 50 nM BIO capped molars of animals treated with Clodrosome, in comparison to the mineral content of the control animals (Encapsome). Unpaired t-test analysis: MTA **P = 0.0018; 50 nM BIO in CS ***P = 0.0003. 100 μm scale bars. *D* dentine, *RD* reparative dentine.

In order to test the effect of macrophage depletion on dentine repair we implemented a macrophage depletion method using clodronate liposomes^6^. In this method, macrophages ingest clodronate liposomes and undergo cell death, therefore modifying the abundance of macrophages^21^. To confirm whether the depletion was working, mice spleens were analysed whilst the teeth were decalcifying. Immunofluorescence and flow cytometry of the spleen 1, 5, and 14 days after dental damage showed a clear decrease in F4/80+ cells (40% decrease), confirming that the clodronate liposome protocol was targeting macrophage reduction in the animal body (Supplementary Fig. 2). Damaged teeth were then analysed to verify the effect of modification of the macrophage population on reparative dentine formation (Fig. 1). Both the control and clodronate-treated animals were collected at 2 and 4 weeks after damage, having undergone 4 (2 weeks) and 6 injections during the reparative window (post damage). Histological analysis of damaged molars from Clodrosome treated animals revealed reduction and phenotypically sparser reparative dentine secreted when compared to control, in both pulp capping treatments (MTA and 50 nM BIO in CS) (Fig. 1A–D, Supplementary Fig. 3). Radiological images and Mineral formation analysis using μCT comparing the treatment group (Clodrosome) and control group (Encapsome) showed that animals treated with Clodrosomes produced significantly less reparative dentine than controls (average 30–40% less; MTA **P = 0.0018; 50 nM BIO in CS ***P = 0.0003) (Fig. 1A′–D′, E). This highlighted that macrophages are cells contributing to reparative dentine formation, and when reduced during repair, they significantly impair reparative dentine formation capacity.

### Neutrophils in dentine repair

**Figure 2:**
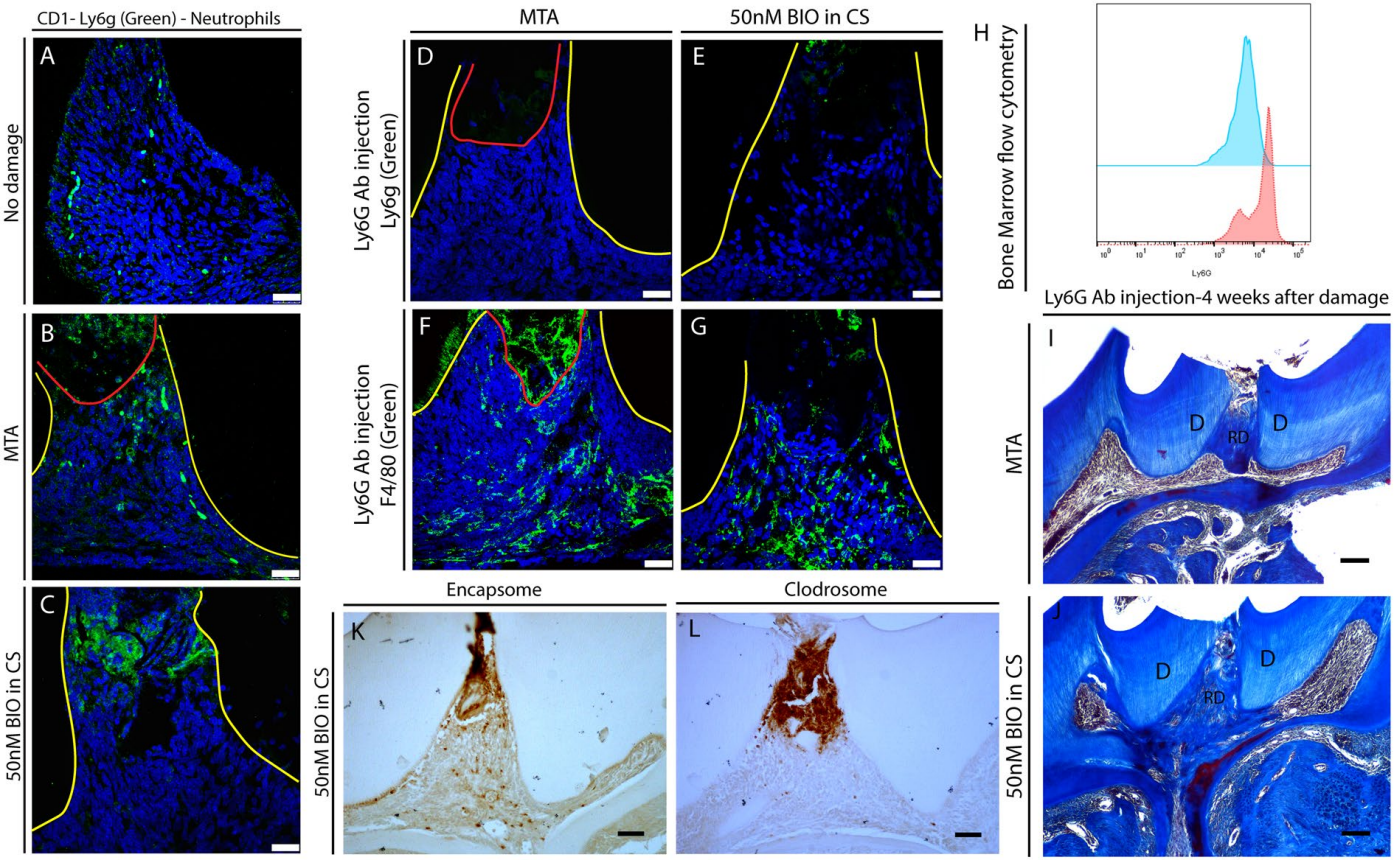
Neutrophils in repair. (**A**–**C**) Immunofluorescence for Ly6G in CD1 wild-type upper first molar. (**A**) Non-damaged molars did not show resident Ly6G+ cells in the dental pulp. (**B**, **C**) Damaged molars capped with either MTA or 50 nM BIO in CS, and collected 1 day after damage presented Ly6G+ neutrophils at damage site. (**D**–**G**) Ly6G antibody injected mice 1 day after damage. (**D**, **E**) MTA or 50 nM BIO in CS capping showed reduced Ly6G+ cells at the molar damage site following neutrophil depletion. (**F**, **G**) F4/80 + macrophages were present in neutrophil depleted dental pulp. (**H**) Flow cytometry for Ly6G in bone marrow of mice femur (Ly6G antibody treated [blue] and non-treated [red]) showed clear decrease of Ly6G + cell number. (**I**, **J**) 4 weeks after capping—Ly6G Antibody treated CD1 wild-type. Neutrophil depleted molars capped with either MTA or 50 nM BIO in CS capping showed robust reparative dentine secretion. (**K**, **L**) Macrophage depletion effect on neutrophils. Immunohistochemistry for Ly6G in damaged upper first molars, capped with 50 nM BIO in CS and injected with Clodrosome or Encapsome, showed increase of Ly6G + neutrophils at damage site when macrophages are depleted. Fluorescence: 25 μm scale bars; Yellow line delineates dental pulp—red line delineates MTA area; immunohistochemistry & histology: 100 μm scale bars.

Having shown that macrophage reduction affects reparative dentine formation, we investigated whether neutrophil reduction affects dentine repair formation. Immunohistochemical staining showed that dental pulp did not show resident Ly6G+ neutrophils (Fig. 2A). However, after dental damage, both MTA and 50 nM BIO capped molars showed Ly6G+ neutrophils at the damage site (Fig. 2B,C). To better understand the effect of neutrophil depletion on dentine repair, we neutralised neutrophils using Ly6g antibody injections^22^. Although we detected macrophages at the damage site, immunohistochemical staining did not show neutrophils 1 day after damage (Fig. 2D–G). To confirm the effectiveness of Ly6g antibody treatment, mouse bone marrow aspirated from femurs was analysed by flow cytometry. The treatment decreased 41% of Ly6G+ cells in the bone marrow 1 day after antibody injections (Fig. 2H).

To investigate reparative dentine secretion after neutralising antibody treatment of neutrophils in vivo, treated and control damaged molars were allowed to repair for 4 weeks. Neutropenic animals had increased reparative dentine secretion and vital pulp, suggesting that decrease of neutrophils supressed inflammation in the dental pulp, leading to increased reparative dentine secretion (Fig. 2I,J). Interestingly, following macrophage depletion with clodronate liposomes, we observed an increase of Ly6G+ neutrophils at the damage site, suggesting that absence of macrophages may create excessive inflammation at the damage site (Fig. 2K,L). Together, these results suggest that there is a link between inflammation control and macrophage presence in the dental pulp.

### Macrophages and Wnt responsive stem cells in the dental pulp

**Figure 3:**
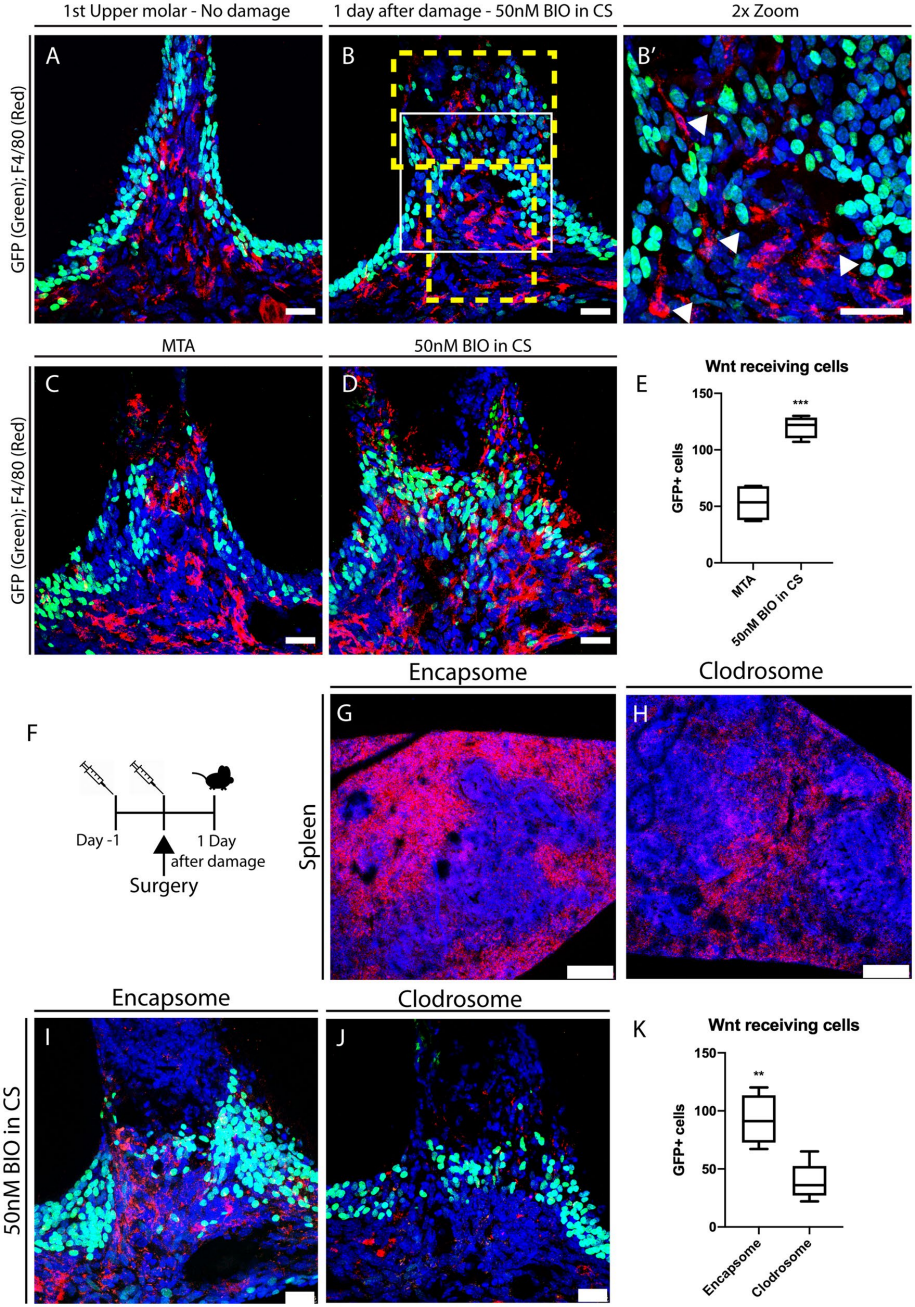
F4/80+ macrophages and Wnt responsive cells activity. (**A**) TCF/Lef:H2B-GFP mice upper first molar with no damage shows Wnt responsive cells (Green) relation to F4/80+ macrophages (Red). (**B**, **B′**) One day after damage in TCF/Lef:H2B-GFP mice upper molars capped with 50 nM BIO in CS showed that F4/80+ macrophages (Red) do not colocalise with Wnt responsive cells (Green), however they are adjacent to Wnt receiving cells (arrow heads). (**C**–**E**) Comparison of TCF/Lef:H2B-GFP mice upper molars capped with either MTA or 50 nM BIO in CS showed that there was a significant increase in Wnt responsive cells at the damage site when teeth were caped with a GSK-3 inhibitor small molecule, increasing physical proximity with F4/80+ macrophages (Red). (**F**) Time frame of Clodrosome and Encapsome injection in TCF/Lef:H2B-GFP mice. Injections (syringe) and collection date (mouse). (**G**, **H**) Spleen sections of TCF/Lef:H2B-GFP mice injected with either Clodrosome or Encapsome and immunohistochemistry for F4/80 showing decrease of macrophage population in the spleen. 250 μm scale bars. (**I**–**K**) One day after damage in TCF/Lef:H2B-GFP mice upper molars capped with 50 nM BIO in CS, injected with Clodrosome or Encapsome. Immunofluorescence for F4/80+ macrophages (Red) and GFP+ cells (Green) showed decrease of macrophages and Wnt receiving cells numbers at the damaged dental pulp when mice were injected with Clodrosome*.* (**B**) Yellow dashed line delineates areas in the pulp where cells were counted. Unpaired t-test analysis: (**E**) ***P = 0.0004. (**I**–**K**) **P = 0.0038. (**A**–**D**) 75 μm scale bars, (**G**–**J**) 25 μm scale bars.

Having shown that macrophages are important for tooth repair and dentine formation we aimed to understand further the impact of macrophages on repair, by investigating whether Wnt receiving stem cells are affected in the pulp as observed in gut crypts^16^. We used a Wnt pathway activator to evaluate the Wnt receiving stem cells response in the dental pulp, and assayed the effect of macrophage depletion on Wnt receiving dental pulp stem cells in vivo. Using the reporter mouse line TCF/Lef:H2B-GFP, we observed that F4/80+ macrophages and Wnt receiving cells (GFP+) were physically closely located in the pulp but the signal was not co-localising as the same cell (Fig. 3A,B,B′). Therefore, dental pulp macrophages were not acting as Wnt receiving cells.

Next, we investigated the interaction between macrophages and Wnt receiving stem cells. We initially confirmed that a significant increase (approximately 25%) of Wnt receiving cells in the pulp at the damage site was seen when molars were capped with the Wnt agonist, in comparison to MTA capped molars (*P = 0.0146) (Fig. 3C–E). Further, to understand the impact of macrophages on Wnt receiving stem cells in the dental pulp, we injected TCF/Lef:H2B-GFP reporter mice with Clodrosome (Fig. 3F). Immunofluorescence for F4/80 in the spleen showed a clear decrease of macrophage numbers (Fig. 3G,H). Immunofluorescence for GFP and F4/80 in damaged molars showed that macrophage depletion resulted in a significant decrease (30%) in the number of Wnt receiving stem cells in the dental pulp (Fig. 3I–K).

Together, these results demonstrate that Wnt receiving cells and macrophages (resident and transient) interact with each other, and furthermore, the presence of macrophages in the dental pulp is essential for Wnt receiving stem cell activation.

### Wnt elevation affects macrophage polarisation in the dental pulp

Our results established that the Wnt receiving cells and macrophages in the dental pulp have an interactive relationship during dentine repair. The M1 to M2 macrophage polarisation is an important feature of tissue repair and thus we investigated the effect of Wnt pathway activation on macrophage polarisation in vivo.

**Figure 4:**
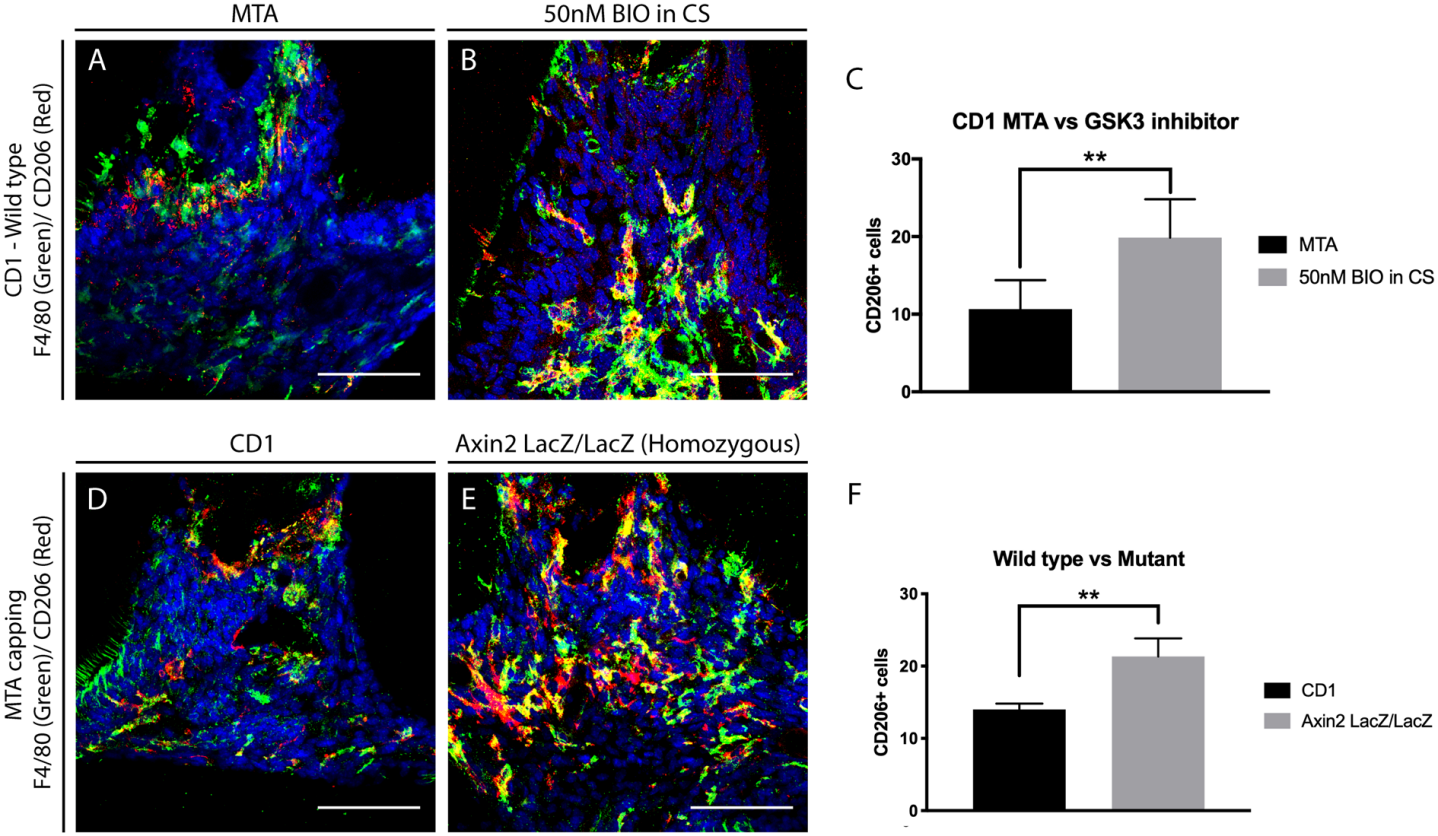
Anti-inflammatory macrophages in dentine repair. (**A**–**C**) Comparison of anti-inflammatory M2 macrophages 1 day after damage in CD1 wild type mice upper first molars capped with either MTA or 50 nM BIO in CS. Staining for F4/80 (Green—panel marker) and M2 marker CD206 (Red) showed significantly fewer colocalised macrophages in MTA capped molars when compared to molars capped with 50 nM BIO in CS. (**D**–**F**) To confirm the results with the different capping materials, CD1 wild type mice and *Axin2*^LacZ/LacZ^ were damaged and capped with MTA. One day after damage, the number of M2 macrophages were analysed. Staining for F4/80 and M2 marker CD206 confirmed increase of M2 macrophages at the damage site when there is increase of Wnt activity. (**C**) Unpaired t-test analysis: **P = 0.0033; (**F**) **P = 0.0014. 75 μm scale bars.

To verify M1–M2 polarization, double labelling immunofluorescence for F4/80 and CD206 (anti-inflammatory M2 macrophages) in damaged molars capped with Wnt activator were studied. A 40% increase in double F4/80+; CD206+ was observed in Wnt activated teeth compared with MTA controls (Fig. 4A–C), suggesting that Wnt activation produces an anti-inflammatory effect in the dental pulp. To confirm the effect of Wnt elevation on polarisation of macrophages in the dental pulp we used *Axin2*^−LacZ/LacZ^ mice to investigate the effect of increased activation of Wnt activity in the dental pulp. Molars of mice with continuous elevation of the Wnt pathway (*Axin2*^−LacZ/LacZ^) showed significant increase of M2 macrophages at the damage site (**P = 0.0014), compared to controls (Fig. 4D–F).

Polarisation from M1 to M2 is conducted by TGF-β^23–27^, therefore, further confirmation was done by investigating TGF-β1 expression and apoptosis in the dental pulp. Our results showed that Wnt activator treated molar significantly upregulated TGF-β1 in the dental pulp 1 day after damage when compared to MTA capped molars (Supplementary Fig. 4). In addition, TUNEL staining shows decrease of apoptotic cells at the damage site when GSK-3 inhibitor small molecules are used to cap the dental pulp (Supplementary Fig. 5). Together, these findings suggest that Wnt elevation in dental pulp cells produces an acceleration in the polarisation from pro-inflammatory to anti-inflammatory stage of the macrophages early in the reparative process, however since macrophages are not themselves Wnt-responsive this effect must be indirect.

## Discussion

The aim of dental treatments in cavitated teeth is to restore dental functionality and maintain dental pulp vitality. Following a dental intervention, the host biological response to treatment is to secrete tertiary dentine. In deep cavities, reparative dentine is formed and Neves et al.^20^ and Zaugg et al.^28^ showed that Wnt activation via GSK-3 antagonists enhances biologic reparative dentine repair in dental cavities. Reparative dentine formation, however, is a complex process that requires a mild inflammatory response within the tooth^29,30^. Therefore, in order to demonstrate the biological effect of this novel translational approach, we investigated the impact of pharmacological Wnt/β-catenin elevation on the host dental pulp inflammatory response.

Babb, et al.^31^ showed that Wnt receiving cells are stem cells capable of differentiating into odontoblast-like cells in an in vivo damage scenario. However, Wnt receiving stem cells in the gut crypt do not differentiate when macrophages are depleted^16^. Our results show that macrophages are also key players in the dentine reparative process and uncoupling the immune reaction via depletion of macrophages significantly decreases the amount of Wnt responsive cells and increases neutrophil accumulation at the damage site. Together, that leads to an increased inflammatory reaction, resulting in a decrease in reparative dentinogenesis^32,33^. Since neutrophil depletion did not eliminate macrophages in the dental pulp following damage and reparative dentine capacity increased, these results suggest that macrophages are key cells for regulation of local dental pulp stem cell activation and inflammatory balance in tertiary dentinogenesis.

Next, we carried out experiments to understand M1 to M2 polarisation, a transition that is a key step in tissue repair^24,27,34–38^. Predominance of M2 macrophages signals an advanced phase in tissue repair^19,24^. Inflammatory host-response modulation affects reparative capacity, and in our small molecule model we saw an acceleration of M1 to M2 macrophage polarisation 1 day after damage, compared to the controls. This accelerated polarisation was confirmed in *Axin2*^LacZ/LacZ^ mice showing a significant increase of M2 macrophages (CD206+) at the damage site, suggesting that host Wnt elevation leads to an anti-inflammatory effect.

M2 macrophages have been described as having subtypes according to their function^19,24^. M2a and M2c macrophages are characterised by mannose receptor expression and their polarisation was found to be driven by TGF-β expression^19^. Our GSK-3 antagonist model increased TGF-β1 expression post damage and decreased apoptotic cells at the damage site, aligning with previously described M1–M2 polarisation literature^24^. Since TGB-β1 is not essential for odontoblast differentiation during repair, its primary role may be in modulating macrophage activity^39^. Therefore, increase of Wnt pathway in the dental pulp indirectly stimulates polarisation of M2 macrophages in vivo.

Previous studies suggested that stem cells indirectly modulate the immune response to an anti-inflammatory stage in situ^37,38^. Since we see an increase of Wnt responsive stem cells in the dental pulp when Wnt agonist small molecules are used for capping and an increase of macrophages adjacent to Wnt receiving cells, a molecular interaction may be taking place between the two cell types^16^. This notion is also supported by evidence shown here that pharmacological ablation of macrophages leads to a decrease in Wnt responsive cells in the pulp.

When a tooth is cavitated and the dental pulp becomes exposed, it opens a doorway for bacterial colonisation and infection. Therapeutic approaches to avoid infection and stimulate repair are currently based on synthetic inorganic materials such as inert calcium silicate-based material MTA. However, we have previously shown that biological activation of the Wnt/β-catenin signalling via GSK-3 antagonist small molecules reparative dentinogenesis is superior to MTA^20^. Here, we demonstrate the biological modulatory effect of this novel approach. Therefore, by taking a “host biological response to therapy development” axis for a novel translational approach, we demonstrated that Wnt activation in the dental pulp results in a host enhanced capacity to repair dentine damage with exposed dental pulp.

Overall, here we show that Wnt activation via GSK-3 antagonist small molecules results in an improved reparative capacity due to two main factors: (1) direct activation and increase in number of Wnt receiving stem cells at the damage site, and (2) indirect modulation of macrophages to an anti-inflammatory state from M1 to M2. Together these results suggest that pharmacological host modulation via Wnt agonist small molecules has biological potential to be used in future dental therapies.

## Materials and methods

### Mouse lines

All animals used in this study were handled in accordance with UK Home Office Regulations project license 70/7866 and personal license I6517C8EF, approved by the KCL animal ethics committee and comply with ARRIVE guidelines. Experimental procedures were approved by the King’s College Ethical Review Process. CD1 (Wild-type) were obtained from the New Hunts House Biological Services Unit (NHH-BSU) (n = 152), TCF/Lef:H2B-GFP reporter mice were a kind gift from Anna-Katerina Hadjantonakis (n = 20), and *Axin2*-LacZ/LacZ mice were obtained from the Jackson Laboratory (n = 4). Mice were collected at E12.5 (n = 2) and E14.5 (n = 2) for embryonic analysis, and 6 weeks old mice were collected either at control time point (No Surgery), post 1, 3, 5 days or 4 weeks after damage.

### Damage protocol with pulp exposure

Pulp exposure and damage was performed as previously described^20^, and MTA or collagen sponges soaked in 50 nM BIO were used to cap the upper first molars post dental pulp exposure.

### Macrophage depletion

CD1 wild-type and TCF/Lef:H2B-GFP reporter mice mice were injected with a macrophage depletion kit Clodrosome and Encapsome (Encapsula NanoSciences). Clodrosome is a Liposomal Clodronate, and Encapsome is the Control Liposomes. For the CD1 wild-type, the animals were injected 200 μl of either solution intraperitoneally. An injection was given 1 day prior to the surgery day, mice were injected again on the first day after damage, third day after damage, fifth day after damage, seventh day after damage, 14th day after damage, and 21st day after damage (7 injections overall). The animals were collected 1, 5, 14 (n = 3 per treatment, group, and time point) and 28 days after damage (n = 4 per treatment and group). A total of 52 animals were used for this experiment.

For the TCF/Lef:H2B-GFP reporter mice, an injection was given 1 day prior to the surgery day, then the mice were injected again on the day of the surgery and collected 1 day after damage. A total of six animals were used for this experiment (n = 3 per treatment).

### Neutrophil depletion

CD1 wild-type mice were injected intraperitoneally with a neutrophil depletion kit *InVivoPlus* anti-mouse Ly6G (Bio X Cell), diluted in *InVivoPlus* pH 7.0 Dilution Buffer (Bio X Cell). The animals were injected with an initial dose of 400 μg of antibody 2 h before surgery, then after every 3 days 100 μg of antibody was injected. The animals were collected on the first day after damage and 28 days after damage (n = 3 per treatment and timepoint). In total the animals collected at the 28 days after damage received ten injections A total of 12 animals were used for this experiment.

### μCT analysis

μCT analysis was performed as previously described^20^. Mice maxillae were collected, fixed in PFA 4% overnight at 4 °C, and scanned using a Bruker Skyscan1272 μCT scanner. Microview software programme (GE) was used for visualisation and analysis. Two-dimensional images were obtained from μCT cross-sectional images of the superior first molar to evaluate mineral formation. Three-dimensional (3D) reconstructions were used to verify mineral content analysis. To assay tissue mineral content a Region of Interest (ROI) of X = 0.2 mm, Y = 0.4 mm, and Z = 0.2 mm was set as standard for all the samples and the mineral analysis was performed. The region measured comprised only of the damaged site. ROI complete filled with mineral = 0.017 mg/cm^3^.

### Histology

After 2 weeks of decalcification in 19% ethylenediaminetetraacetic acid (EDTA) pH 6, the teeth were embedded in wax blocks and sectioned at 8-μm thickness. Sections were stained using Masson’s trichrome or Hematoxylin and Eosin.

### Immunohistochemistry

PFA 4% fixed spleen cryosections were used to perform immunofluorescence. For mouse molars, after 2 weeks of decalcification in 19% ethylenediaminetetraacetic acid (EDTA) pH 6, the teeth were sectioned. Two PBS washes and 0.2%PBT impermeabilization, the sections were blocked and incubated with chicken polyclonal anti–green fluorescent protein (anti-GFP) antibody (1:300; Abcam, ab13970), Rat monoclonal Anti-Ly6g (1:200; Abcam, ab25377), Rat monoclonal Anti-F4/80 (1:200; Abcam, ab6640), and Rabbit polyclonal Anti-Mannose Receptor (CD206) (1:200; Abcam, ab64693)overnight at 4 °C. Sections were washed and exposed to appropriate secondary antibody [Alexa-Fluor 488 goat anti-chicken IgY (H + L) 1:250, Alexa-Fluor 488 goat anti-rat, IgG (H + L) 1:250, Alexa-Fluor 568 goat anti-rat, IgG (H + L) 1:250, Alexa-Fluor 568 goat anti-rabbit IgG (H + L) 1:250, Biotinylated Anti-Rat IgG (H + L)] for 1 h RT. For the immunohistochemistry ImPACT DAB Peroxidase (HRP) Substrate was used. Nuclear counter staining was done with Hoechst (1:1000), or Erhlich’s hematoxylin. The manual cell counting was performed using the built-in cell-counter plugin of the ImageJ program.

### Flow cytometry

The spleen and bone marrow aspirate from immunocompromised mice were placed in SB Buffer (1%FBS, 10 mM HEPES buffer pH7, PBS), the cells were then isolated by centrifugation and prepared for cytometry analysis. Following suspending the cells in cold SB buffer, 0.1–10 μg/ml of conjugated primary antibody (Alexa Fluor 488 anti-mouse Ly-6G/Ly-6C (Gr-1), Biolegend, 108417; APC anti-mouse F4/80, Biolegend, 123116) diluted in 3% BSA/PBS was added and incubated for 30 min in the dark at RT^40^. The cells were then washed and resuspended in cold PBS, 10% FCS, 1% sodium azide. Just before analysis, 1 μl of DAPI was added to the samples and flow cytometry analysis was performed.

## Supplementary information

Supplementary Information.
