# Supplementary material for: Macrophage modulation of dental pulp stem cell activity during tertiary dentinogenesis
Source: Sci Rep. 2020 Nov 19;10:20216. doi: 10.1038/s41598-020-77161-4 (PMC7678850; doi:10.1038/s41598-020-77161-4)
Supplement: Supplementary file 1 — Supplementary Information. [file 41598_2020_77161_MOESM1_ESM.docx]

**Macrophage modulation of dental pulp stem cell activity during tertiary dentinogenesis**

Vitor CM Neves^1,2^, Val Yianni^1^, Paul T Sharpe^1^

1 Centre for Craniofacial and Regenerative Biology, Faculty of Dentistry, Oral & Craniofacial Sciences, Kings College London, UK

2 Centre for Host-Microbiome Interactions, Faculty of Dentistry, Oral & Craniofacial Sciences, Kings College London, UK

Corresponding address : Prof. Paul T Sharpe ([paul.sharpe@kcl.ac.uk](mailto:paul.sharpe@kcl.ac.uk))

Centre for Craniofacial and Regenerative Biology, Faculty of Dentistry, Oral & Craniofacial Sciences, Floor 27, Guy’s Tower, Guy’s Hospital, SE1 9RT, London, UK.

**Supplementary Figure**

**Supplementary Figure 1 - Macrophage presence in the dental organ in homeostasis and repair**

**(A-B)** Frontal section of mouse embryo at the level of the first upper molar during odontogenesis. Macrophages (F4/80+ cells), were present at the mesenchyme during bud stage (white dashed line – epithelium budding). At cap stage there was an increase of Macrophages in the mesenchyme of the developing tooth (orange dashed line – epithelium folding). **(C)** Non-damaged CD1 upper first molars (yellow dashed line – delineates the dental pulp). Homeostatic dental pulp showed abundance of resident F4/80+ macrophage. **(D-I)** Immunofluorescence for F4/80 in CD1 wild-type upper first molar damaged and capped with either MTA or 50nM BIO in CS after one, three and five days from damage. MTA and 50nM BIO capped dental pulps presented F4/80+ cells recruited to the damage site, remaining in the area during the early reparative time (First five days). (yellow dashed lines – delineates the dental pulp, * damage site). 25μm scale bars.


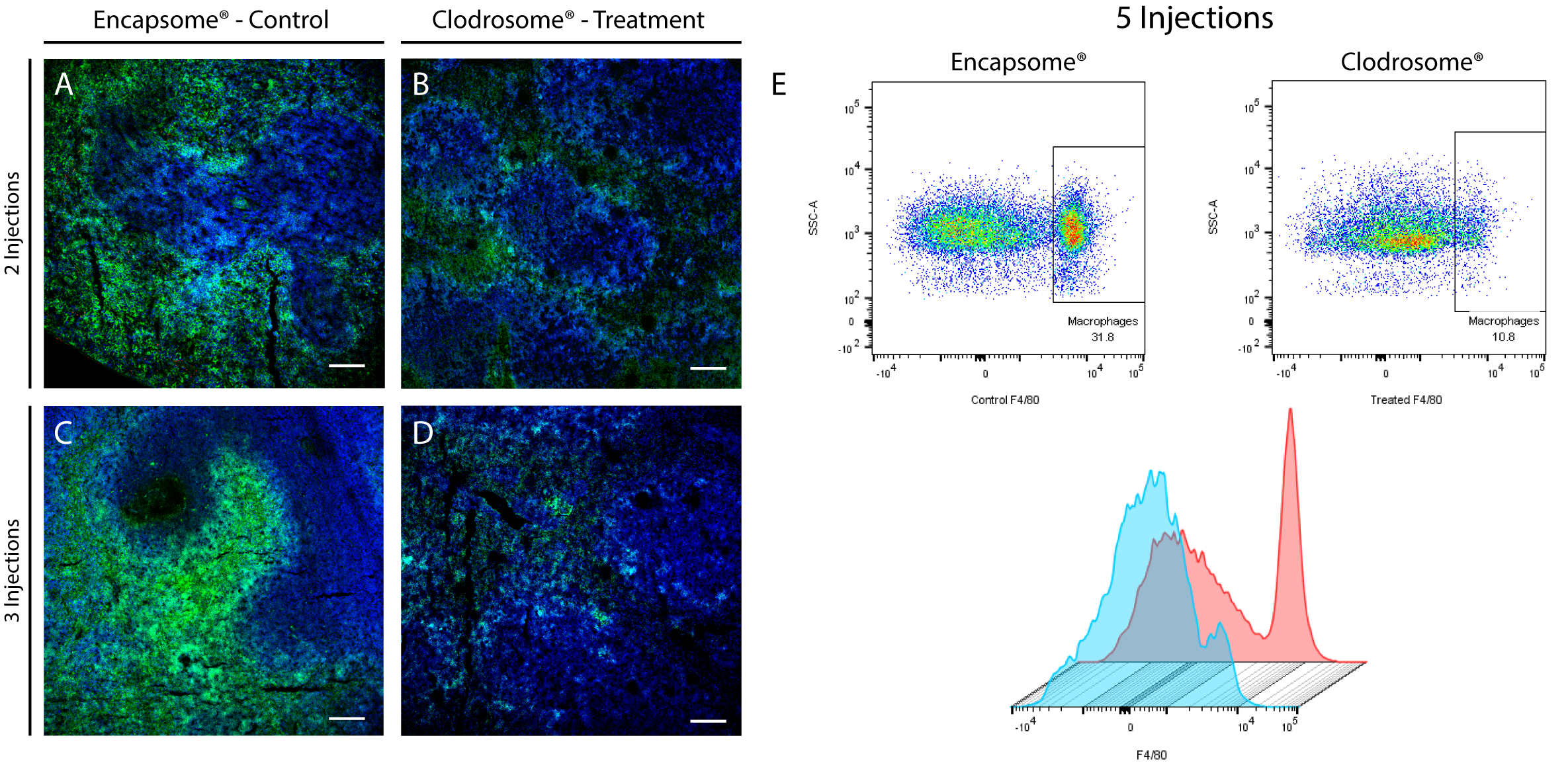
**Supplementary Figure 2 Macrophage depletion in the spleen**

**(A)** 1 day after damage mouse spleen stained for F4/80 (Green) showed positive cells in the organ, whereas samples that had Clodrosome injected showed very few F4/80+ cells **(B)**. The same was seen in spleens of mice collected 5 days after damage **(C,D)**. **(E)** Flow cytometry for F4/80 showed a drastic decrease of F4/80+ cells in the spleen 2 weeks after damage. 75μm scale bar.


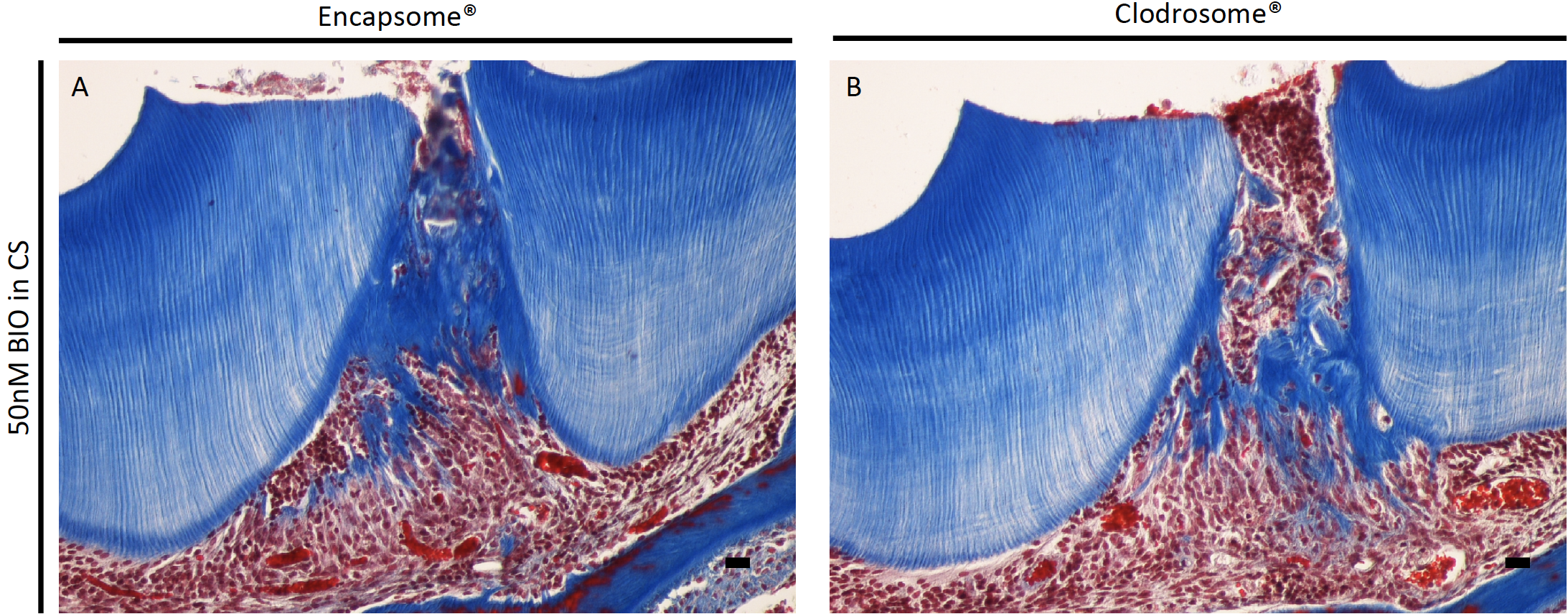


**Supplementary Figure 3 – 2 weeks time point Macrophage depletion**

Masson trichrome staining of molars capped with 50nM BIO in CS post 2 weeks of damage having gone through either Clodrosome or Encapsome injections. The reparative dentine of mice injected with Clodrosome was affected showing sparse reparative dentine secreted.


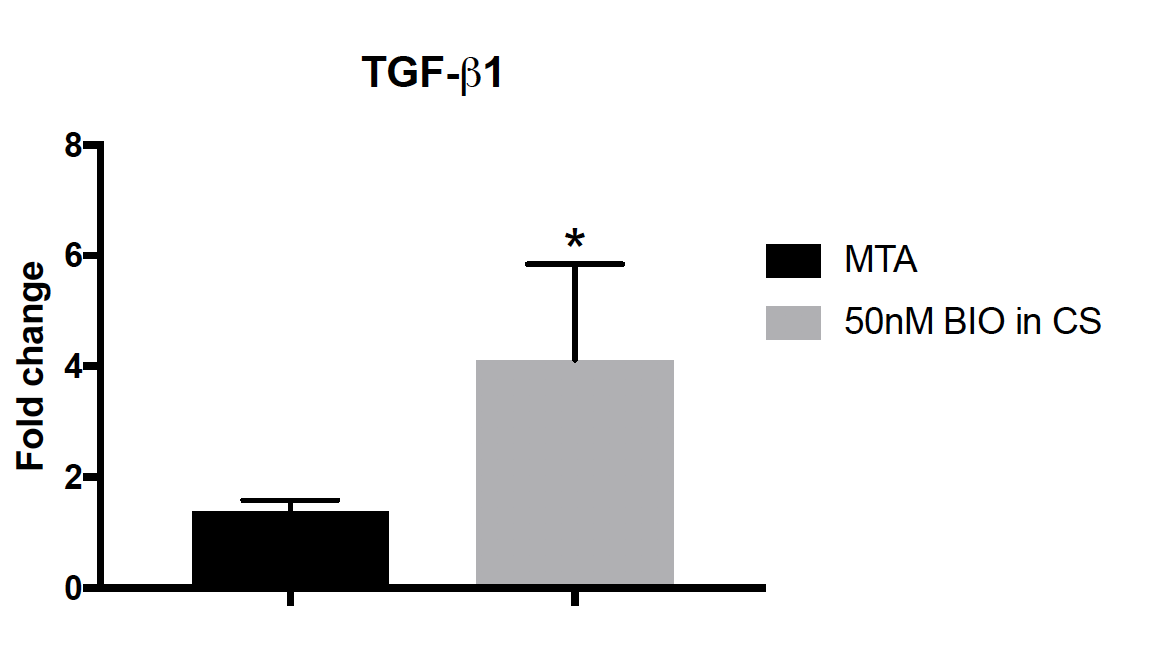


**Supplementary figure 4. In vivo dental pulp expression of TGF-β1**

TGF-β1 qPCR for dental pulp cells collected after one day from damage (using non-damaged dental pulp as control), capped with either MTA or BIO. Gene expression analysis showed significant difference on TGF-β1expression one day after damage. (Unpaired t-test analysis: TGF-β1 BIO *P=0.0448)


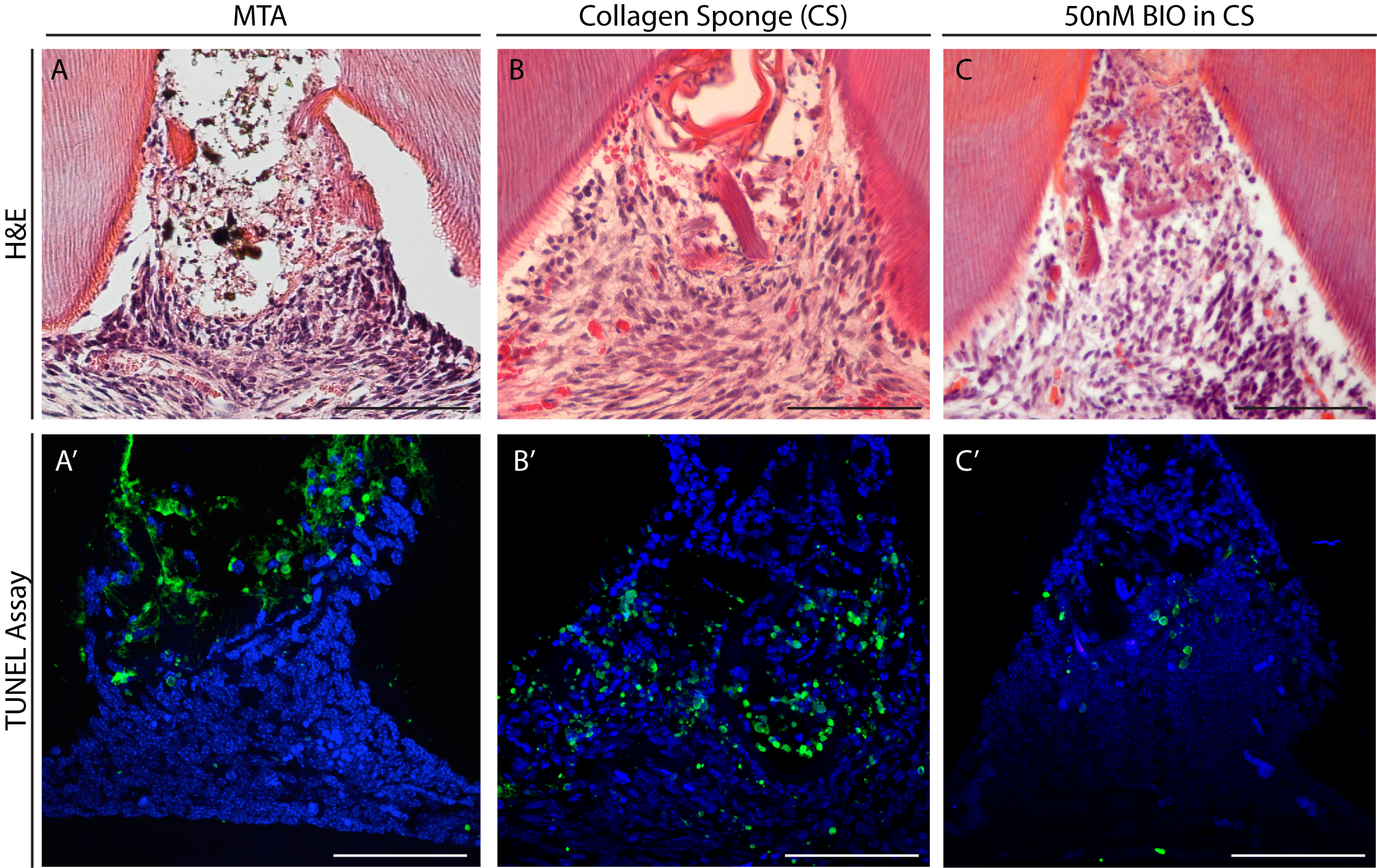


**Supplementary figure 5. Apoptosis 1 day after damage**

Sagittal sections of upper first molars at the damage site. 1 day after damage in CD1 wild type mice. (A-C) H&E histology showed no sign of necrosis in any capping technique (MTA, CS, 50nM BIO). Dentine debris and inflammatory cells were present in the damage site of all groups. TUNEL assay showed apoptosis of cells in contact with MTA (A’). (B’) Collagen sponge capping showed high rate of apoptosis happening in the dental pulp. (C’) 50nM BIO capped molars showed low rate of apoptosis in the dental pulp. 75μm scale bar. Green marks for apoptotic cells. Blue is nuclei staining.(n=3 per treatment)

**Supplementary Materials and Methods**

**qPCR**

RNA was extracted from the dental pulp collected from CD1 P21 wild-type mice without injury (control) and 1 day after injury treated with either MTA or 50nM BIO in CS, using TRIzol (Thermo Fisher Scientific) as recommended by the manufacturer. A total of 45 animas were used in this experiment (n=1 requires 5 animals or 10 teeth for RNA extraction). The RNA was quantified using Nanodrop and reverse transcribed into cDNA. Beta-actin was used as housekeeping gene (Forward- GGCTGTATTCCCCTCCATCG, Reverse- CCAGTTGGTAACAATGCCTGT) and used to normalise the gene of interest TGF-β1 (Forwards- CTGCTGACCCCCACTGATAC, Reverse- AGCCCTGTATTCCGTCTCCT). Expression and fold change calculated in Excel between treatments and controls by ΔCq and ΔΔCq. The graphs were plotted with GraphPad Prism, and so was the Unpaired t-test statistical analysis.

**TUNEL**

The TUNEL assay was performed using ApopTag Peroxidase In Situ Apoptosis Detection Kit (EMD Millipore) as described by the manufacturer, and color was developed using TSA (Perkin Elmer NEL701A001) 488 fluorophore 1:100. The sections were counterstained with Hoechst nucleic acid stain (1:10,000; Thermo Fisher Scientific).
